# Development and evaluation of a rapid and simple diagnostic assay for COVID-19 based on loop-mediated isothermal amplification

**DOI:** 10.1371/journal.pntd.0008855

**Published:** 2020-11-04

**Authors:** Rokusuke Yoshikawa, Haruka Abe, Yui Igasaki, Saeki Negishi, Hiroaki Goto, Jiro Yasuda

**Affiliations:** 1 National Research Center for the Control and Prevention of Infectious Diseases (CCPID), Nagasaki University, Nagasaki, Japan; 2 Department of Emerging Infectious Diseases, Institute of Tropical Medicine (NEKKEN), Nagasaki University, Nagasaki, Japan; 3 Canon Medical systems, Otawara, Tochigi, Japan; 4 Graduate School of Biomedical Sciences, Nagasaki University, Nagasaki, Japan; WRAIR, UNITED STATES

## Abstract

Severe acute respiratory syndrome coronavirus 2 (SARS-CoV-2) is a highly pathogenic novel coronavirus that has caused a worldwide outbreak. Here we describe a reverse transcription loop-mediated isothermal amplification (RT-LAMP) assay that uses a portable device for efficient detection of SARS-CoV-2. This RT-LAMP assay specifically detected SARS-CoV-2 without cross-reacting with the most closely related human coronavirus, SARS-CoV. Clinical evaluation of nasal swab samples from suspected SARS-CoV-2 pneumonia (COVID-19) patients showed that the assay could detect over 23.7 copies within 15 min with a 100% probability. Since the RT-LAMP assay can be performed with a portable battery-supported device, it is a rapid, simple, and sensitive diagnostic assay for COVID-19 that can be available at point-of-care. We also developed the RT-LAMP assay without the RNA extraction step–Direct RT-LAMP, which could detect more than 1.43 x 10^3^ copies within 15 min with a 100% probability in clinical evaluation test. Although the Direct RT-LAMP assay was less sensitive than the standard RT-LAMP, the Direct RT-LAMP assay can be available as the rapid first screening of COVID-19 in poorly equipped areas, such as rural areas in developing countries.

## Author summary

Rapid, accurate and simple diagnostic assay is required to provide an appropriate treatment and establish a surveillance system to prevent the spread of COVID-19. In this study, we developed the RT-LAMP assay using a portable platform equipped with a battery for detection of SARS-CoV-2 in clinical specimens. It could detect over 23.7 copies within 15 min. Since RNA extraction step is one of the main obstacles for rapid diagnosis, we also developed the RT-LAMP assay without this step, Direct RT-LAMP. This assay could detect over 203 copies of viral RNA in virus-spiked swab solution within 10.5 min. Since our RT-LAMP assay is a rapid, simple and highly sensitive diagnostic system for identifying COVID-19 case, it would be useful for the detection of SARS-CoV-2, especially in the field or the settings with insufficient infrastructure.

## Introduction

In December 2019, an outbreak of pneumonia caused by a novel coronavirus, severe acute respiratory syndrome coronavirus 2 (SARS-CoV-2), was first reported in Wuhan, China [1,2]. SARS-CoV-2 has rapidly spread worldwide, and the World Health Organization (WHO) has declared the corona virus disease (COVID-19) as a pandemic on 11 March 2020 [3]. Until the day of the announcement, over 4,962,707 confirmed cases of SARS-CoV-2 pneumonia in at least 200 countries of all continents, except Antarctica, had been reported [4].

COVID-19 is usually characterized by fever, cough, and myalgia or fatigue, and shows a mortality of 3.4% with progressive respiratory failure or multiple organ failure [5]. The median incubation period of COVID-19 is 5.1 days in 50 provinces, regions, and countries outside Wuhan, Hubei province, China [6]. However, several reports described that SARS-CoV-2 may show a longer incubation period (~19 days) and human-to-human transmission in the incubation period, making difficult to prevent the infection from spreading [7]. The transmission mechanism of SARS-CoV-2 has not been fully elucidated, although it shows a highly communicable ability through aerosol droplets [8]. Currently, early detection of SARS-CoV-2 and adequate isolation of patients are the most effective countermeasures to prevent human-to-human transmission of the virus. Therefore, development of an on-site, rapid, and sensitive diagnostic assay for SARS-CoV-2 infections is of top priority to provide an appropriate treatment and establish a surveillance system to prevent the spread of COVID-19.

SARS-CoV-2 infection is diagnosed in the laboratory mainly using nucleic acid amplification tests (NAATs). Several reverse transcription-quantitative PCR (RT-qPCR) assays were developed by WHO soon after the report of genome sequences of SARS-CoV-2, and are currently the gold standard to detect virus-specific RNA [9]. However, RT-qPCR requires a step of viral RNA extraction prior to the test and the use of a qPCR thermal cycler instrument connected to stable electric power. Moreover, there is potential risk of viral RNA degradation during sample transport to the laboratory due to inappropriate storage conditions. Thus, novel diagnostic technologies that can be conducted at the point-of-care, including rural areas of developing countries in which stable electricity may be unavailable, are crucial to control and monitor SARS-CoV-2 infections.

Reverse transcription loop-mediated isothermal amplification (RT-LAMP) is a rapid and sensitive RNA detection method performed under simple isothermal conditions using four or six target sequence-specific oligonucleotide primers [10]. Since LAMP reactions can be performed using a battery-driven portable equipment, RT-LAMP assay is suitable for on-site diagnosis even in insufficiently equipped facilities. We have previously developed RT-LAMP assays for Ebola and Zika viruses in response to recent outbreaks of associated diseases, and the assays have been deployed for field surveillance in Guinea and Brazil, respectively [11–13]. Here, we report an RT-LAMP assay specific for SARS-CoV-2 with a comparable sensitivity to standard RT-qPCR. This assay shows a potential to be performed without the RNA extraction step, representing a rapid and simple diagnostic NAAT.

## Materials and methods

### Ethics statement

This study was approved by the Institutional Review Boards of Nagasaki University (approval no. 200409234).

### Viral RNA and synthesized standard RNA

Viral RNA of SARS-CoV-2 and SARS-CoV were kindly provided by the Japanese National Institute of Infectious Diseases (NIID), and Dr. Koichi Morita from the Nagasaki University, Japan, respectively. To calculate the copy number of viral genome detection in both LAMP and quantitative PCR (qPCR) assays, synthesized RNAs were prepared using T7 RiboMAX Express Large Scale RNA Production System (Promega) with the artificially synthesized DNA of the viral target sequence conjugated with the T7 promoter sequence.

### LAMP primer design

LAMP primers for SARS-CoV-2 detection were designed based on the sequences of the ORF1b region of the virus. The SARS-CoV-2 sequences available in GenBank and GISAID were aligned using BioEdit 7.0.5.3 software (http://www.mbio.ncsu.edu/BioEdit/bioedit.html) to identify conserved regions. A consensus sequence of the ORF1b region was used to design LAMP primers through Primer Explorer V5 software (Eiken; http://primerexplorer.jp/). The RT-LAMP assay requires a set of 6 primers: 2 outer primers (F3 and B3), 2 inner primers (FIP and BIP), and 2 loop primers (LF and LB). FIP consists of a complementary sequence of F1 and a sense sequence of F2, whereas BIP includes a complementary sequence of B1 and a sense sequence of B2 (10). The detailed primer sequences used for SARS-CoV-2 amplification are shown in Table 1.

**Table 1:** Sequences of LAMP primers.

Name	Type	Sequence (5’–3’)
LAMP_ORF1b-1_F3	F3	AACCTGAGTTTTATGAGGCT
LAMP_ORF1b-1_B3	B3	TCCTAAGTAAAGTTGAGTCACA
LAMP_ORF1b-1_FIP	FIP	TGCAAGCACCACATCTTAATGAAGTCGCATACAGTCTTACAGGCT
LAMP_ORF1b-1_BIP	BIP	ACGACCATGTCATATCAACATCACAACATCACAACCTGGAGCAT
LAMP_ORF1b-1_LF	LF	CAAAGAACACAAGCCCCAAC
LAMP_ORF1b-1_LB	LB	GTCTTGTCTGTTAATCCGTATGTTTG
LAMP_ORF1b-2_F3	F3	GGTTTTTTCACTTACATTTGTGG
LAMP_ORF1b-2_B3	B3	TCCTCCAAAATATGTAATTTGCA
LAMP_ORF1b-2_FIP	FIP	GCGAAGTGTCCCATGAGCTTATAAACTAGCTCTTGGAGGTTCCG
LAMP_ORF1b-2_BIP	BIP	AATGCGTCATCATCTGAAGCATTTTCATAACCATCTATTTGTTCGCG
LAMP_ORF1b-2_LF	LF	TCAGCATTCCAAGAATGTTCTGT
LAMP_ORF1b-2_LB	LB	ATTGGATGTAATTATCTTGGCAAACC

### RT-LAMP assay

RT-LAMP was performed with Isothermal Master mix reagent ISO-004 (Canon medical systems) using the Genelyzer FIII real-time fluorescence detection platform (Canon medical systems). The reaction mixture (total volume = 25 μL) contained 15 μL of Isothermal Master Mix, 1 U of AMV Reverse Transcriptase (Nippon gene), 20 pmol (each) of FIP and BIP primers, 5 pmol (each) of F3 and B3 outer primers, 10 pmol (each) of F and B loop primers, and 5 μL of RNA sample (template). The reaction was performed at the following conditions: for the ORF1b-1 primer set, 68°C for 20 min, followed by a dissociation analysis at 95°C–75°C with the temperature change rate of 0.1°C/s; for the ORF1b-2 primer set, 67°C for 20 min, followed by a similar dissociation analysis. Extracted RNA from samples or heat inactivated swab samples were used as a template. Synthesized RNAs containing the target sequence of the LAMP assay were used as positive controls. Nonspecific amplification was excluded by comparing the melting temperature to that of the positive control [13].

### RT-qPCR assay

RT-qPCR was performed using the One Step PrimeScript III RT-qPCR Mix (Takara Bio) as reported previously [14]. The reaction mixture (total volume = 20 μL) contained 10 μL of 2× One Step PrimeScript III RT-qPCR Mix, 0.4 μL of ROX Reference Dye, 2 μL of 10× primer-probe mixture, 2 μL of RNA sample, and 5.6 μL of RNase-free water. Two different sets of primer-probe mixture were used in this study. The first set was developed by NIID (NIID-qPCR) and used with the final concentration of 500 nM, 700 nM, and 200 nM of the forward primer, reverse primer, and FAM-labeled probe, respectively [15]. The second primer-probe set was reported by WHO for detection of E gene of SARS-CoV-2 and SARS (WHO-qPCR), and used 400 nM of each primer and 200 nM of the probe [9]. The NIID-qPCR reaction was performed using the StepOnePlus instrument (Applied Biosystems) with a thermal cycle program of 52°C for 5 min, 95°C for 10 sec, followed by 45 cycles of 95°C for 5 sec, and 60°C for 30 sec WHO-qPCR was performed with the same program. Cut-off values were set at the threshold cycle (Ct) value of 40. To quantify viral RNA, a standard curve was generated with 10-fold serial dilutions of synthesized standard RNA of the qPCR target sequences.

### Validation of the assays using clinical specimens

A validation study was performed using 224 nasal swab samples collected in Nagasaki prefecture, Japan, without any clinical information. For RT-LAMP and RT-qPCR, RNA was extracted using QIAamp Viral RNA Mini Kit (QIAGEN) according to the instructions of the manufacturer. For Direct RT-LAMP, the samples were heated at 95°C for 10 min for inactivation of SARS-CoV-2. Aliquots of 5 μL of RNA samples or heat inactivated swab samples were used for the LAMP assay with ORF1b-1 primer set and RT-qPCR with NIID primer set.

## Results

### Sensitivity of RT-LAMP assay using in vitro synthesized RNA

We designed two SARS-CoV-2 specific LAMP primer sets that targeted conserved sequences in the ORF1b region (name: ORF1b-1 and ORF1b-2) (Table 1). To examine the sensitivity of the RT-LAMP assay for SARS-CoV-2, serial 10-fold dilutions of in vitro synthesized RNA possessing the target sequence of Wuhan-Hu-1 strain were used as template. All (6/6) or 83% (5/6) of the reactions with 50 copies of RNA yielded positive results with ORF1b-1 (RT-LAMP/ORF1b-1) or ORF 1b-2 primer sets (RT-LAMP/ORF1b-2), respectively (Table 2). Additionally, RT-LAMP/ORF1b-1 could detect samples containing 5 RNA copies once in six replicates. Moreover, both RT-LAMP/ORF1b-1 and RT-LAMP/ORF1b-2 could detect 500 RNA copies within 8 min and 50 RNA copies within 11.8 min (Table 2). These results indicate that this RT-LAMP assay can be used as a rapid and sensitive test for the detection of SARS-CoV-2 RNA.

**Table 2:** Sensitivity and detection time of RT-LAMP assay.

ORF1b-1				
Copies/reaction	500	50	5	Water
Time (min)	7.5	9.0	-	-
	7.3	8.8	10.5	-
	8.0	11.8	-	-
	6.8	9.3		-
	6.8	9.0	-	-
	7.3	8.5	-	-
Average	7.3	9.4	-	-
SD	0.4	1.1	-	-
Positive	6/6	6/6	1/6	0/6
ORF1b-2				
Copies/ reaction	500	50	5	Water
Time (min)	8.0	11.8	-	-
	7.8	-	-	-
	7.3	11.5	-	-
	7.5	9.0	-	-
	7.3	9.5	-	-
	6.8	10.3	-	-
Average	7.4	10.4	-	-
SD	0.4	1.1	-	-
Positive	6/6	5/6	0/6	0/6

### Specificity of RT-LAMP assay

Following this, to examine the specificity of the RT-LAMP assay, we performed the assay using RNAs extracted from SARS-CoV, which is the most closely related human coronavirus to SARS-CoV-2, and SARS-CoV-2 (n = 2 for each viral RNA). The RT-LAMP did not detect SARS-CoV RNA regardless of the primer set used, while SARS-CoV-2 RNA was successfully detected with both primer sets (Table 3). We confirmed the presence of SARS-CoV RNA using RT-qPCR with WHO primer (Table 3, Ct = 27.5 (7,780 copies) and 27.7 (6,890 copies) in duplicated samples). These results indicate that our RT-LAMP assay is highly specific for SARS-CoV-2.

**Table 3:** Specificity of RT-LAMP assay.

	Primer set	SARS		SARS-CoV-2
RT-LAMP(min)	ORF1b-1	-	-	+ (11.5)
	ORF1b-2	-	-	+ (9.75)
RT-qPCR (Ct)	WHO	+ (27.5)	+ (27.7)	

### Sensitivity comparison between RT-LAMP assay and RT-qPCR assay

To compare the sensitivity of RT-LAMP/ORF1b-1, RT-LAMP/ORF1b-2, and RT-qPCR assays using the NIID (RT-qPCR/NIID) or the WHO (RT-qPCR/WHO) primer sets, we prepared 10-fold serial dilutions of viral RNA extracted from SARS-CoV-2 JPN/TY/WK-521 strain (GISAID: EPI-ISL-408667). All RT-LAMP and RT-qPCR assays could detect the viral RNA in all samples of dilutions lower than 10^−4^ (Table 4). The average detection time of RT-LAMP assays was 12.8 min. The Ct values of the RT-qPCR/NIID and RT-qPCR/WHO for 10^−4^ dilution samples were 37.75±0.21 and 35.81±0.19, which corresponded to 15.9±2.2 and 26.7±4.0 copies per reaction, respectively (Table 4). In addition, RT-qPCR/NIID and RT-qPCR/WHO assays detected the 10^−5^ dilution of the viral RNA samples twice and once in 3 replicates, respectively. Furthermore, the RT-LAMP/ORF1b-1 assay also detected the 10^−5^ virus dilution once in 3 replicates; although, the RT-LAMP/ORF1b-2 failed to detect these samples (Table 4). The 10^−5^ dilution was estimated to include 5.6 copies in RT-qPCR/NIID and 2.6 copies in RT-qPCR/WHO (Table 4). Taken together, these results indicate that the RT-LAMP/ORF1b-1 assay has almost the same sensitivity as RT-qPCR assay, which is a highly sensitive detection method.

**Table 4:** Comparison of sensitivity between RT-qPCR and RT-LAMP assays.

			Dilution rate of viral RNA				
			10^−2^	10^−3^	10^−4^	10^−5^	Water
RT-LAMP	ORF1b_1	Time (min)	6.5	7.8	9.0	-	-
			6.3	7.3	9.0	9.5	-
			6.5	7.8	12.8	-	-
		Average	6.4	7.6	10.3	-	-
		SD	0.1	0.2	1.8	-	-
		Positive	3/3	3/3	3/3	1/3	0/3
	ORF1b_2	Time (min)	6.8	7.5	9.5	-	-
			6.0	7.8	10.8	-	-
			6.8	7.5	10.5	-	-
		Average	6.5	7.6	10.3	-	-
		SD	0.4	0.1	0.5	-	-
		Positive	3/3	3/3	3/3	0/3	0/3
RT-qPCR	NIID	Ct value	30.93	34.08	37.59	39.08	-
			30.79	34.12	38.05	39.34	-
			30.60	33.79	37.61	-	-
		Average	30.77	34.00	37.75	39.21	-
		SD	0.14	0.15	0.21	0.13	-
		copies/ reaction	2036.9	214.4	17.5	6.1	-
			2249.6	208.6	12.7	5.0	-
			2577.0	264.4	17.3	-	-
		Average	2287.8	229.1	15.9	5.6	-
		SD	222.1	25.0	2.2	0.5	-
		Positive	3/3	3/3	3/3	2/3	0/3
	WHO	Ct value	29.31	32.80	35.96	38.93	-
			29.23	32.72	35.94	-	-
			29.41	32.79	35.55	-	-
		Average	29.32	32.77	35.81	38.93	-
		SD	0.07	0.03	0.19	0.00	-
		copies/ reaction	3503.2	254.9	23.7	2.6	-
			3694.3	269.0	24.1	-	-
			3246.6	256.5	32.4	-	-
		Average	3481.4	260.2	26.7	2.6	-
		SD	183.5	6.3	4.0	0.0	-
		Positive	3/3	3/3	3/3	1/3	0/3

### Evaluation of RT-LAMP assay without RNA extraction step

In most cases, genome analysis for detection of RNA viruses requires a step of RNA extraction from clinical specimens. Since this step usually takes about 30 min, it is one of the main obstacles for rapid diagnosis. Therefore, the feasibility of the RT-LAMP assay without RNA extraction for clinical specimens was evaluated using human nasal and oral mixed swab suspensions spiked with SARS-CoV-2 JPN/TY/WK-521 strain. We prepared the nasal and oral mixed swab samples with 10-fold serial dilutions of SARS-CoV-2. These mixed swab samples were heated at 95°C for 10 min for inactivation of SARS-CoV-2. We conducted RT-LAMP/ORF1b-1 and RT-qPCR/NIID assays with the heat inactivated swab samples, which we named “Direct RT-LAMP” and “Direct RT-qPCR,” respectively. Simultaneously, we also conducted RT-LAMP/ORF1b-1 and RT-qPCR/NIID assays using RNAs extracted from the nasal and oral mixed swab samples spiked with SARS-CoV-2 as reference tests. Sample viral dilutions ranging from 10^−2^ to 10^−6^ dilution were detected within 10.5 min in both RT-LAMP and Direct RT-LAMP (Table 5). Furthermore, both Direct RT-LAMP and RT-LAMP could detect 10^−7^ diluted virus samples with 33% probability; nevertheless, RT-qPCR could efficiently detect these samples. In this swab spike tests, both assays have same sensitivity. However, the detection time for Direct RT-LAMP is longer than that of RT-LAMP (mean 0.75 min). Therefore, composition of swab samples may affect the efficiency of genome amplification in RT-LAMP assay. Standard RT-qPCR using extracted RNA indicated that 10^−6^ and 10^−7^ dilution include 203 ± 21.4 and 12.8 ± 4.78 copies of viral RNA per reaction, respectively. Taken together, these results suggest that Direct RT-LAMP assay can be used as a rapid and sensitive diagnostic test for SARS-CoV-2.

**Table 5:** Evaluation of RT-LAMP assay without RNA extraction step.

			Dilution rate of virus						
	Template		10^−2^	10^−3^	10^−4^	10^−5^	10^−6^	10^−7^	Water
Direct RT-LAMP	Swab solution	Time (min)	5.5	6.0	6.8	7.5	10.5	-	-
			5.3	6.0	6.8	7.3	9.0	10.5	-
			5.5	6.0	7.3	7.5	9.0	-	-
		Average	5.4	6.0	6.9	7.4	9.5	-	-
		SD	0.1	0.0	0.2	0.1	0.7	-	-
		Positive	3/3	3/3	3/3	3/3	3/3	1/3	0/3
RT-LAMP	RNA	Time (min)	4.3	5.0	5.8	6.5	7.3	-	-
			5.5	6.0	7.3	7.5	9.0	-	-
			4.8	5.8	6.3	6.8	7.5	9.5	-
		Average	4.8	5.6	6.4	6.9	7.9	-	-
		SD	0.5	0.4	0.6	0.4	0.8	-	-
		Positive	3/3	3/3	3/3	3/3	3/3	1/3	0/3
Direct RT-qPCR	Swab solution	Ct value	23.14	26.47	29.86	33.43	36.40	-	-
			23.22	26.50	29.87	33.29	36.93	-	-
			23.01	26.46	29.75	33.28	36.96	-	-
		Average	23.12	26.48	29.83	33.34	36.76	-	-
		SD	0.09	0.02	0.05	0.07	0.26	-	-
		copies/ reaction	1.14× 10^6^	9.07×10^4^	6.92×10^3^	4.62×10^2^	4.87×10^1^	-	-
			1.06× 10^6^	8.84×10^4^	6.89×10^3^	5.12×10^2^	3.25×10^1^	-	-
			1.25× 10^−6^	9.15×10^4^	7.50×10^3^	5.17×10^2^	3.17×10^1^	-	-
		Average	1.15×10^6^	9.02×10^4^	7.10×10^4^	4.97×10^2^	3.77×10^1^	-	-
		SD	7.73×10^4^	1.30×10^3^	2.81×10^2^	2.49×10^1^	7.85×10^0^	-	-
		Positive	3/3	3/3	3/3	3/3	3/3	0/3	0/3
RT-qPCR	RNA	Ct value	20.89	24.29	27.76	31.13	34.70	38.95	-
			20.97	24.34	27.80	31.18	34.35	37.66	-
			20.75	24.28	27.65	31.00	34.51	38.16	-
		Average	20.87	24.30	27.74	31.10	34.52	38.26	-
		SD	0.09	0.03	0.07	0.07	0.14	0.53	-
		copies/ reaction	6.23×10^6^	4.75×10^5^	3.41×10^4^	2.64×10^3^	1.77×10^2^	7.03×10^0^	-
			5.85×10^6^	4.56×10^5^	3.29×10^4^	2.55×10^3^	2.29×10^2^	1.87×10^1^	-
			6.93×10^6^	4.77×10^5^	3.72×10^4^	2.92×10^3^	2.04×10^2^	1.28×10^1^	-
		Average	6.33×10^6^	4.69×10^5^	3.47×10^4^	2.71×10^3^	2.03×10^2^	1.28×10^1^	-
		SD	4.45×10^5^	9.44×10^4^	1.78×10^3^	1.56×10^2^	2.14×10^1^	4.78×10^0^	-
		Positive	3/3	3/3	3/3	3/3	3/3	3/3	0/3

### Evaluation of RT-LAMP assay using clinical specimens

To evaluate the diagnostic accuracy of RT-LAMP and Direct RT-LAMP, we analyzed 224 nasal swab samples from suspected COVID-19 patients using these experimental protocols at the same time has RT-qPCR/NIID as reference test. Sixteen samples were suspended in Hank's Balanced Salt Solution (HBSS) and the remaining samples were suspended in phosphate-buffered saline (PBS) solution.

**Fig 1:**
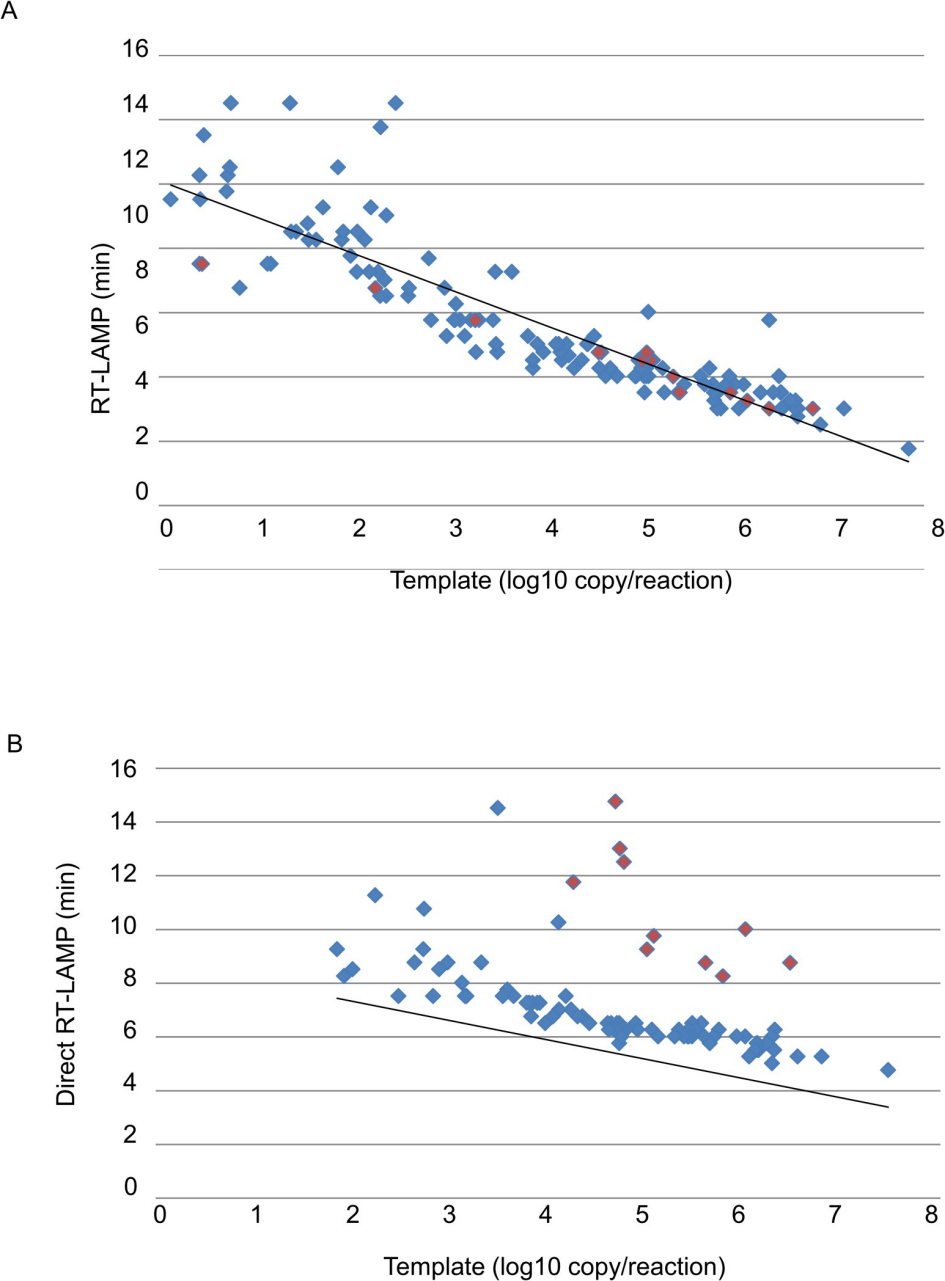
Detection time of SARS-CoV-2 RNA from clinical specimens with the RT-LAMP and Direct RT-LAMP assays. Detection time of clinical samples determined by RT-LAMP/ORF1b-1 (A) and Direct RT-LAMP/ORF1b-1 (B) were plotted to viral RNA copy numbers per reaction with reference RT-qPCR/NIID. The samples suspended by PBS or HBSS are shown as blue or red dots, respectively.

As shown in Fig 1, the detection time of RT-LAMP and Direct RT-LAMP was significantly correlated with copy number of SARS-CoV-2 RNA (Peason’s correlation (r) = -0.25 and -0.22, for RT-LAMP and Direct RT-LAMP, respectively). This correlation indicates that viral RNA copies in clinical samples could be roughly estimated from the detection time of RT-LAMP assay.

**Fig 2:**
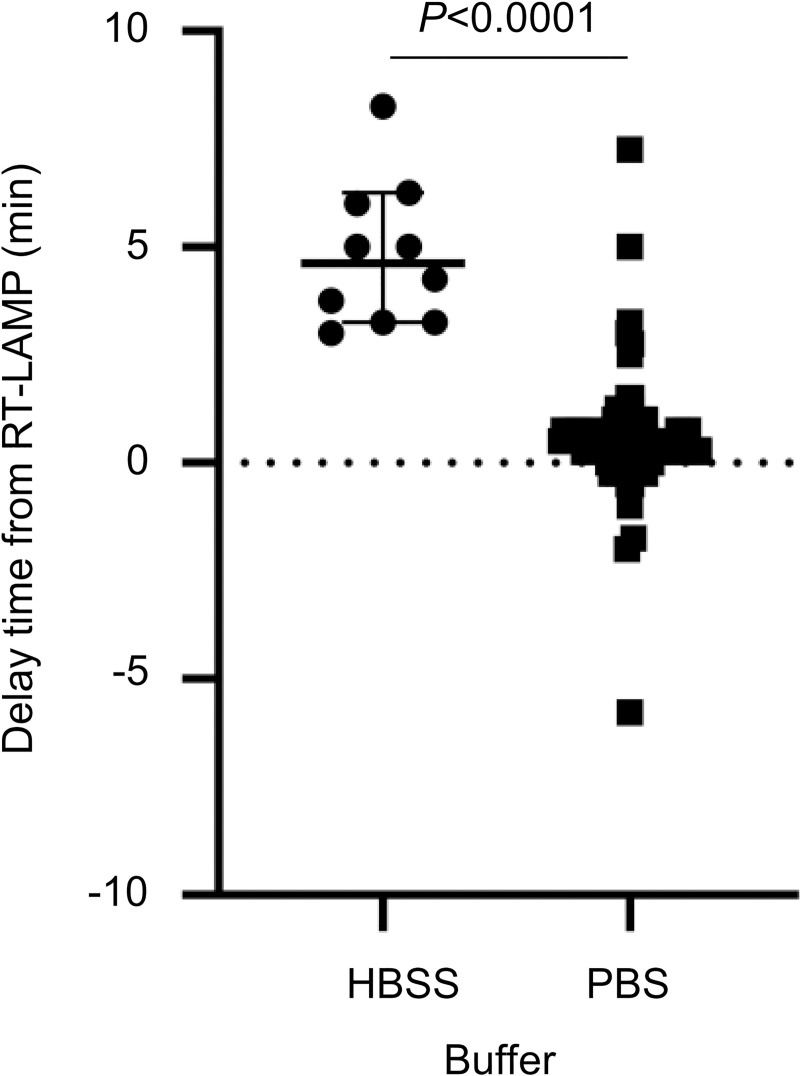
Comparison of detection time between RT-LAMP and Direct RT-LAMP. The delay time of detection by Direct RT-LAMP is shown for nasal swabs suspended in HBSS and PBS.

The detection time for Direct RT-LAMP using PBS-suspended samples PBS was delayed on average by only 0.45 min compared with RT-LAMP using extracted RNA (Fig 2). In contrast, the detection time of Direct RT-LAMP using HBSS-suspended swab samples was 3 to 8 min (mean 4.8 min) later than that of RT-LAMP using extracted RNA (Fig 2), suggesting that HBSS is not appropriate as swab sample suspension solution for Direct RT-LAMP. Therefore, the results of the 16 samples suspended in HBSS were excluded from the analysis (Table 6).

**Table 6:** Diagnostic accuracy of the RT-LAMP assay and the Direct RT-LAMP assay for SARS-CoV-2 compared with the reference RT-qPCR assay.

			RT-LAMP			Direct RT-LAMP		
		Copies/reaction	Positive	Negative	Sensitivity	Positive	Negative	Sensitivity
RT-qPCR	Positive	>10^7^	2	0	100%	1	0	100%
		10^6^−10^7^	17	0	100%	14	0	100%
		10^5^−10^6^	24	0	100%	17	0	100%
		10^4^−10^5^	18	0	100%	21	0	100%
		10^3^−10^4^	18	0	100%	16	1	94%
		10^2^−10^3^	18	0	100%	9	8	53%
		10^1^−10^2^	12	2	86%	2	18	10%
		10^1^>	10	5	67%	0	19	0%
	negative		15	67				

A total of 126 samples were deemed positive by the reference RT-qPCR assay. In this evaluation test, the RNA amount of each clinical sample was estimated to be in the range from 1.3 to 6.88 x 10^7^ copies per reaction. The RT-LAMP/ORF1b-1 assay detected all clinical samples that had more than 23.7 copies of viral RNA as positive for SARS-CoV-2 with 100% probability (Table 6 and S1 Table). Detection probabilities of the assay were 50% for 10–19.8 copies and 67% for less than 10 copies. All positive results were obtained within 14.5 min (Fig 1A and S1 Table). The Direct RT-LAMP with ORF1b-1 primer set detected samples that had more than 1.43 x 10^3^ copies of viral RNA with 100% probability (Table 6 and S2 Table). The assay still showed 53% and 10% probabilities for 10^2^−10^3^ and 10^1^−10^2^ viral copies, respectively. The minimum copy numbers which were possible to detect was 70.8 copies (S2 Table). For samples that had 7.08 x 10^1^−1.41 x 10^3^ viral copies, composition of swab samples seem to affect the results; although, the results were consistent with those from Direct RT-LAMP for swab samples spiked with the virus (Table 5). Therefore, it should be carefully noted that low viral RNA copy numbers may not be detected by this Direct RT-LAMP assay depending on the composition of the sample. And, this Direct RT-LAMP assay cannot be used to prove a negative result for SARS-CoV-2 infection.

## Discussion

In this study, we developed a rapid and sensitive diagnostic assay for SARS-CoV-2 infections, which is based on RT-LAMP. The assay could detect 23.7 copies of viral RNA within 15 min from clinical specimens (Fig 1A, Table 6, and S1 Table). Currently, RT-qPCR is the gold standard assay of COVID-19 diagnosis and takes at least 2–3 hours to complete all processes including RNA extraction followed by a quantitative PCR step using a real-time thermal cycler. To shorten the assay time, several research groups developed RT-LAMP assays against SARS-CoV-2 [16–21]; however, the detection sensitivity and assay time can be improved. Huang et al. reported a novel RT-LAMP assay that could detect 2 copies of viral RNA after an additional gel electrophoresis step, requiring more assay time than the normal RT-LAMP method [21]. In addition to RT-LAMP, we also developed a remarkable rapid assay, Direct RT-LAMP, that does not require the extraction of viral RNA from clinical specimens. This assay could detect 203 copies of viral RNA in the virus-spiked swab solution within 10.5 min (Table 5). The LAMP method has an excellent specificity accomplished by 6 to 8 different primer binding regions in the target DNA sequence. Nevertheless, several viruses require multiple sets of primers to detect whole lineages/genotypes due to genetic diversity of their genome sequences. Lassa virus (LASV) has 6 lineages and has highly diverse genome even within the same lineage. There is a single report of a RT-LAMP assay for LASV, and the assay requires 3 sets of primers to detect only lineage II [22]. Zika virus also shows diversity in genome sequences between Asian and African genotypes, requiring the mixture of 2 primer sets to detect all strains in one reaction [13]. Fortunately, SARS-CoV-2 shows lower genetic diversity worldwide, and therefore would be suitable to be diagnosed using the simple RT-LAMP assay with one primer set (S1 Fig).

Before the emergence of SARS-CoV-2, two large outbreaks of novel coronavirus diseases were reported: severe acute respiratory syndrome (SARS) in 2002, and Middle East respiratory syndrome (MERS) in 2012 [23,24]. For the molecular diagnosis for these diseases, RT-PCR and RT-qPCR were used as gold standard assays with an immediate announcement of recommended protocols by WHO. Several years since the emergence of these coronavirus diseases, RT-LAMP assays were developed for faster diagnosis [25,26]. For SARS, Thai et al. developed a RT-LAMP assay which is 100-fold more sensitive than RT-PCR [25] and showed clear linearity between viral titer and detection time, and that could detect SARS-CoV even in RT-PCR negative samples. Similar to SARS, several RT-LAMP assays were developed for the detection of MERS-CoV. Shirato et al. developed a RT-LAMP assay for MERS with a sensitivity comparable to standard RT-qPCR, and with sufficient specificity to distinguish MERS-CoV from 20 other respiratory viruses [26]. These past reports indicate advantages in using RT-LAMP assays to detect coronavirus from clinical specimens with sufficient sensitivity.

A recent clinical study that evaluated respiratory tract specimens of COVID-19 patients with severe and mild symptoms showed the initial viral load of 6.17 and 5.11 log_10_ copies per mL, respectively [27]. Posterior oropharyngeal saliva, which might represent a non-invasive reasonable specimen acceptable by patients, contained 4 to 8 log_10_ copies of viral genome per mL in the first 5 days from the symptom onset [27]. Similarly, experimental SARS-CoV-2 infection of rhesus monkeys showed that the initial viral load in nasal or throat swabs also contained 4 to 8 log_10_ copies of viral genome per mL within the first 5 days [28]. Again, our RT-LAMP assay could detect 23.7 copies of SARS-CoV-2 viral RNA, approximately equivalent to 1,778 copies per mL in clinical specimens, effectively detecting SARS-CoV-2 in samples from patients with both severe and mild symptoms during the acute phase. Whereas, our Direct RT-LAMP is approximately 60-fold less sensitive than RT-LAMP in the assay for clinical specimens, indicating the reliable detection limit of approximately 5 log_10_ copies per mL (Table 6). Thus, although this Direct RT-LAMP assay detects SARS-CoV-2 in high viral titer samples, it should not be used as a sole molecular diagnostic method but used with another assay such as qPCR to avoid false negatives. Nevertheless, Direct RT-LAMP would significantly contribute to the rapid first screening of COVID-19 during an outbreak (1) in the high-risk population (e.g. pregnant women, individuals with a pre-existing illness) who needs an immediate care, and (2) in the resource-limited settings where a centrifuge is unavailable. We also found that HBSS affected the detection time of Direct RT-LAMP (Fig 2), indicating that HBSS contains inhibitors of RT-LAMP amplification. Therefore, in the Direct RT-LAMP assay, it is preferable to suspend swab samples in PBS.

Since the RT-LAMP assay can be performed with a portable battery-driven device, it may be available at point-of-care, even in poorly-equipped settings. Taken together, our results suggest that this new RT-LAMP protocol can be useful as a rapid and sensitive diagnostic assay for COVID-19, and that the Direct RT-LAMP version could also be made available as a much simpler, faster, and low-cost assay. These new assays may contribute to public health control in countries undergoing COVID-19 epidemics, not only for early disease detection, but also to monitor future expansion of viral infections.

## Supporting information

S1 Table The detection time of the RT-LAMP assay in clinical samples of less than 100 copies in Table 6. (DOCX)

S2 Table The detection time of the Direct RT-LAMP assay in clinical samples of less than 10^4^ copies per reaction. (DOCX)

S1 Fig Specificity of LAMP primers. Nucleotide sequences of 40 reference genomes widely selected from each continent, including the Wuhan-Hu-1 strain, are shown for each primer binding site. No mutations were observed in all primer binding sites.
(TIF)
