# Supplementary material for: Development and evaluation of a rapid and simple diagnostic assay for COVID-19 based on loop-mediated isothermal amplification
Source: PLoS Negl Trop Dis. 2020 Nov 4;14(11):e0008855. doi: 10.1371/journal.pntd.0008855 (PMC7668588; doi:10.1371/journal.pntd.0008855)
Supplement: S1 Table — (DOCX) [file pntd.0008855.s001.docx]

**S1 Table.** The detection time of the RT-LAMP assay in clinical samples of less than 100 copies in Table 6

| RT-LAMP |  |  | RT-LAMP | |  |
| --- | --- | --- | --- | --- | --- |
| Time(min) | Copies/reaction |  | | Time(min) | Copies/reaction |
| 10.52 | 84.6 |  | - | | 8.9 |
| 10.27 | 81.6 |  | 8.77 | | 7.0 |
| 12.52 | 74.5 |  | - | | 6.7 |
| 11.27 | 52.0 |  | - | | 6.3 |
| 10.27 | 44.1 |  | 14.52 | | 5.7 |
| 10.27 | 36.9 |  | 12.52 | | 5.5 |
| 10.77 | 36.0 |  | 12.27 | | 5.3 |
| 10.52 | 27.3 |  | 11.77 | | 5.1 |
| 10.52 | 24.2 |  | - | | 3.7 |
| 14.52 | 23.7 |  | - | | 3.5 |
| - | 19.8 |  | 13.52 | | 3.0 |
| - | 17.2 |  | 11.52 | | 2.7 |
| 9.52 | 14.8 |  | 9.52 | | 2.7 |
| 9.52 | 13.7 |  | 12.27 | | 2.7 |
|  |  |  | 11.52 | | 1.3 |

Viral RNA copy numbers in samples were determined by RT-qPCR/NIID.
