# Supplementary material for: Development and evaluation of a rapid and simple diagnostic assay for COVID-19 based on loop-mediated isothermal amplification
Source: PLoS Negl Trop Dis. 2020 Nov 4;14(11):e0008855. doi: 10.1371/journal.pntd.0008855 (PMC7668588; doi:10.1371/journal.pntd.0008855)
Supplement: S2 Table — (DOCX) [file pntd.0008855.s002.docx]

**S2 Table .** The detection time of the Direct RT-LAMP assay in clinical samples of less than 10^4^ copies per reaction

| RT-LAMP |  |  | RT-LAMP |  |  | RT-LAMP |  |
| --- | --- | --- | --- | --- | --- | --- | --- |
| Time(min) | Copies/reaction |  | Time(min) | Copies/reaction |  | Time(min) | Copies/reaction |
| 6.52 | 9.41×10^3^ |  | 8.77 | 9.52×10^2^ |  | - | 9.84×10 |
| 7.27 | 8.27×10^3^ |  | - | 8.88×10^2^ |  | - | 8.92×10 |
| 7.27 | 7.81×10^3^ |  | 8.52 | 7.82×10^2^ |  | - | 8.83×10 |
| 7.27 | 6.99×10^3^ |  | 7.52 | 6.76×10^2^ |  | 8.27 | 8.39×10 |
| 6.77 | 6.78×10^3^ |  | - | 6.05×10^2^ |  | 9.27 | 7.08×10 |
| 7.27 | 6.48×10^3^ |  | 10.77 | 5.48×10^2^ |  | - | 6.79×10 |
| 7.27 | 6.08×10^3^ |  | 9.27 | 5.37×10^2^ |  | - | 6.1×10 |
| 7.52 | 4.49×10^3^ |  | - | 5.25×10^2^ |  | - | 5.10×10 |
| 7.77 | 3.88×10^3^ |  | 8.77 | 4.37×10^2^ |  | - | 5.04×10 |
| 7.52 | 3.49×10^3^ |  | - | 4.16×10^2^ |  | - | 4.32×10 |
| 7.52 | 3.48×10^3^ |  | 7.52 | 3.00×10^2^ |  | - | 3.63×10 |
| 14.52 | 3.10×10^3^ |  | - | 2.85×10^2^ |  | - | 3.50×10 |
| 8.77 | 2.10×10^3^ |  | - | 1.77×10^2^ |  | - | 3.19×10 |
| 7.52 | 1.48×10^3^ |  | 11.27 | 1.73×10^2^ |  | - | 2.23×10 |
| 7.52 | 1.43×10^3^ |  | - | 1.29×10^2^ |  | - | 1.89×10 |
| - | 1.41×10^3^ |  | - | 1.03×10^2^ |  | - | 1.58×10 |
| 8.02 | 1.34×10^3^ |  | 8.52 | 1.02×10^2^ |  | - | 1.54×10 |
|  |  |  |  |  |  | - | 1.17×10 |
|  |  |  |  |  |  | - | 1.04×10 |
|  |  |  |  |  |  | - | 1.03×10 |

Viral RNA copy numbers in samples were determined by RT-qPCR/NIID.
