# Supplementary figures and images for: Development and evaluation of a rapid and simple diagnostic assay for COVID-19 based on loop-mediated isothermal amplification
Source: PLoS Negl Trop Dis. 2020 Nov 4;14(11):e0008855. doi: 10.1371/journal.pntd.0008855 (PMC7668588; doi:10.1371/journal.pntd.0008855)

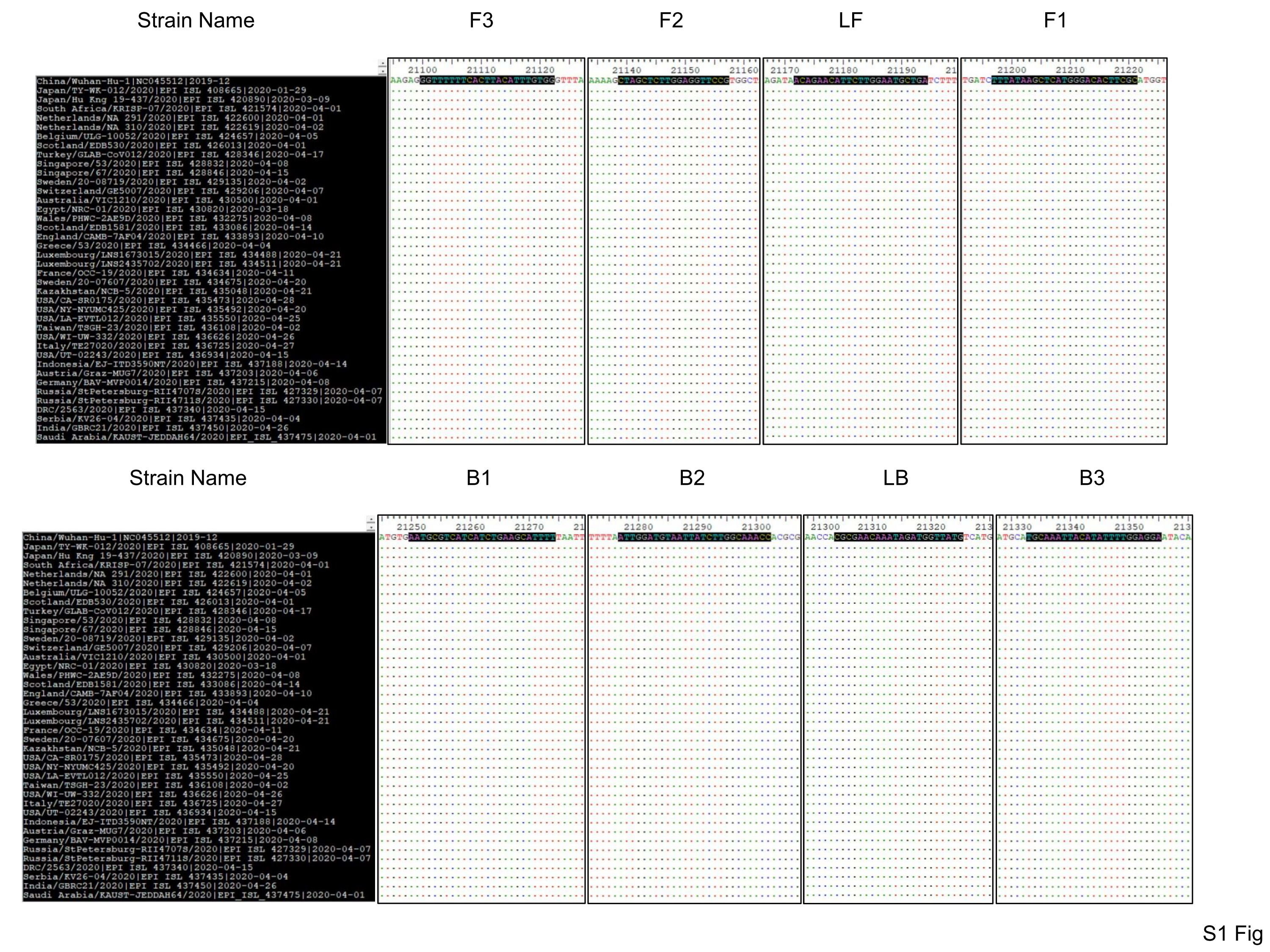

Supplement: S1 Fig — Nucleotide sequences of 40 reference genomes widely selected from each continent, including the Wuhan-Hu-1 strain, are shown for each primer binding site. No mutations were observed in all primer binding sites. (TIF) [file pntd.0008855.s003.tif]
